# Comparison of a Material Point Method and a Galerkin Meshfree Method for the Simulation of Cohesive-Frictional Materials

**DOI:** 10.3390/ma10101150

**Published:** 2017-09-30

**Authors:** Ilaria Iaconeta, Antonia Larese, Riccardo Rossi, Zhiming Guo

**Affiliations:** 1 International Center for Numerical Methods in Engineering (CIMNE), Edificio C1, Campus Norte, Jordi Girona 1-3, 08034 Barcelona, Spain; antoldt@cimne.upc.edu (A.L.); rrossi@cimne.upc.edu (R.R.); 2 Department of Civil and Environmental Engineering (DECA), Technical University of Catalonia (UPC), Edificio C1, Campus Norte, Jordi Girona 1-3, 08034 Barcelona, Spain; guozhiming@live.cn

**Keywords:** particle methods, nonlinear finite element method, implicit MPM, Galerkin Meshfree Method, cohesive-frictional materials

## Abstract

The simulation of large deformation problems, involving complex history-dependent constitutive laws, is of paramount importance in several engineering fields. Particular attention has to be paid to the choice of a suitable numerical technique such that reliable results can be obtained. In this paper, a Material Point Method (MPM) and a Galerkin Meshfree Method (GMM) are presented and verified against classical benchmarks in solid mechanics. The aim is to demonstrate the good behavior of the methods in the simulation of cohesive-frictional materials, both in static and dynamic regimes and in problems dealing with large deformations. The vast majority of MPM techniques in the literatrue are based on some sort of explicit time integration. The techniques proposed in the current work, on the contrary, are based on implicit approaches, which can also be easily adapted to the simulation of static cases. The two methods are presented so as to highlight the similarities to rather than the differences from “standard” Updated Lagrangian (UL) approaches commonly employed by the Finite Elements (FE) community. Although both methods are able to give a good prediction, it is observed that, under very large deformation of the medium, GMM lacks robustness due to its meshfree natrue, which makes the definition of the meshless shape functions more difficult and expensive than in MPM. On the other hand, the mesh-based MPM is demonstrated to be more robust and reliable for extremely large deformation cases.

## 1. Introduction

The numerical simulation of solid mechanics problems involving history-dependent materials and large deformations has historically represented one of the most important topics in computational mechanics. In the framework of discrete mechanics, the Discrete Element Method (DEM) [1] is a popular technique in geotechnical engineering [2] for its advantages in handling large deformation and failure problems and for its relatively easy algorithm and implementation. However, it is well known that time-consuming procedures should be followed to calibrate the material parameters, and it is still extremely hard to handle real scale problems for the huge computational effort required. Within the continuum mechanics framework, a higher number of numerical techniques has been tested, and their effectiveness in the context of cohesive-frictional materials, which undergo large deformations, have been assessed. In this regard, an important contribution is represented by the works of Cervera and coworkers [3,4,5,6], where the Finite Element Method (FEM) is employed to study strain localization in cohesive-frictional materials. However, their work is limited to the case of infinitesimal strains and does not consider the large deformation regime [7]. The Lagrangian viewpoint presents, in this context, a rather obligatory choice, since the adoption of such a framework greatly simplifies the constitutive modeling and the tracking of the entire deformation process. In the case of mesh-based methods, the natural limitation of the Lagrangian approach is related to the deformation of the underlying discretisation, which tends to get tangled as the deformation increases. Aggressive remeshing techniques have proven to be capable of further extending the realm of applicability of Lagrangian approaches, effectively extending the limits of the approach well beyond its original boundaries. For instance, the Arbitrary Lagrangian-Eulerian method (ALE) [8] has been developed in an attempt to overcome the limitation of the Total Lagrangian (TL) and Updated Lagrangian (UL) techniques when severe mesh distortion occurs. Many studies on the application of the ALE method can be found in the literatrue such as, for example, the numerical modeling of friction stir welding [9] or the simulation of geotechnical problems [10]. Another Lagrangian technique, which exploits such remeshing procedures, is represented by the particle finite element method, first developed for the simulation of free surface flows and breaking waves [11,12] and then successfully adapted for structural mechanics problems involving large deformations [13,14,15] or for the simulation of viscoplastic materials [16,17,18,19,20,21] and geomaterials [22,23]. Although the method has broad capabilities, some disadvantages come from the use of remeshing procedures, such as, issues in the parallelization of the code, in the conservation of the mass and difficulties related to the storage of historical variables [24,25,26]. Among the meshfree techniques, it is worth mentioning the Smooth Particle Hydrodynamics method (SPH) [27,28], a Lagrangian meshfree method, initially designed for hydrodynamics problems [29,30]. Recently, this method has been successfully applied to large deformation problems of geomaterials [31,32,33,34]. However, some disadvantages come from its meshfree natrue, such as the impossibility to directly include boundary conditions in the SPH formalism, penetration problems between continua when high speed or impact occurs and the introduction of artificial viscosity to avoid unstable solutions.

Alternative Lagrangian techniques proposed in the literatrue, which solve the drawbacks listed before, are represented by the Material Point Method (MPM) [35,36] and the Galerkin Meshless Method (GMM) [37], which are the objects of study of the current work. The MPM is a particle-based method, extensively used for geotechnical problems [38,39,40] for its capabilities in tracking extremely large deformation while preserving material properties of the material points. Most MPM codes make use of explicit time integrations, due to the ease of the formulation and implementation. However, some implicit versions of MPM can be found in the literatrue. For instance, Guilkey [41] exploits the similarities between MPM and FEM in an implicit solution strategy. Beuth [42] proposes an implicit MPM formulation for quasi-static problems using high order elements and a special integration procedure for partially-filled boundary elements. Finally, Sanchez [43] presented an implicit MPM for quasi-static problems using a Jacobian free algorithm. In the current work, the displacement-based formulation and the time scheme integration of the MPM algorithm are equivalent to those proposed by [41]. The GMM is a truly meshless method, based on a Galerkin formulation. Unlike other methods, such as the element-free Galerkin method [44] or the reproducing kernel particle method [45], the technique employed in this work does not need element connectivity for integration or interpolation purposes. The algorithm presented in [37] to simulate fluid-structrue interaction problems is taken as a starting point and adapted to the simulation of deformable solids.

Both MPM and GMM combine a Lagrangian description of the body under analysis, which is represented by a set of particles, the so-called material points, with the use of a computational mesh: a background grid in the case of MPM and a cloud of nodes in the case of GMM. At each time step, the governing equations are solved on the computational nodes, while history-dependent variables and material information are saved on the particles during the entire deformation process. As MPM and GMM do not employ any kind of remeshing procedure, the calculation is performed always at a local level, allowing an easy adaptation of the code to parallel computation. Moreover, the conservation of the mass is guaranteed during the whole simulation time, as the total mass is distributed between the material points, representing the volume of the entire continuum under study. Last but not least, a remapping of the state variables is avoided, and the employment of complex time-dependent constitutive laws can be used without committing any mapping error. Given the fulfillment of the aforementioned featrues, MPM and GMM could represent suitable choices for the solution of real scale large deformation problems, and a comparison within a unified framework would be beneficial for an objective evaluation of the capabilities of each method.

In the present paper, the reader can find a detailed presentation of the implicit MPM and GMM algorithms and, as the main novelty, an assessment of their performance in terms of accuracy, computational cost and robustness. Both techniques employ a variational Galerkin formulation for solid mechanics problems, defined within an updated Lagrangian kinematic framework, under the assumption of finite strains. The first one uses a fixed background grid, which is deformed and then reset at every solution step. The second one uses a purely Lagrangian meshless approach. The two methods differ conceptually only in the way the shape functions and their gradients are computed. Nevertheless, as we shall see, the use of one technique leads to advantages and disadvantages over the alternative one. Both techniques described are implemented by the authors within Kratos Multiphysics [46,47], a framework for building multi-disciplinary finite element programs in C++.

The paper is structrued as follows: the governing equations are presented in their strong form in Section 2, and the algebraic linearized system is obtained in Section 3. After that, MPM and GMM are briefly revised, and the two algorithms are detailed in Section 4 to emphasize the similarities with an FEM algorithm. Some benchmark tests are simulated to assess the capabilities of the developed formulations (Section 5), and finally, Section 6 provides some concluding remarks and suggestions for futrue research.

## 2. Governing Equations

Let us consider the body B, which occupies a region Ω of the three-dimensional Euclidean space E with a regular boundary ∂Ω in its reference configuration. A deformation of B is defined by a one-to-one mapping:

(1) φ:Ω→E

that maps each point ***p*** of the body B into a spatial point x:

(2) $$x=\varphi \left(p\right)$$

which represents the location of ***p*** in the deformed configuration of B. The region of E occupied by B in its deformed configuration is denoted as $\varphi \left(\Omega \right)$.

The problem is governed by mass and linear momentum balance equations:

(3a) $$\begin{array}{cc} \frac{D\rho }{Dt}+\rho \nabla \cdot v=0\; & \mathrm{in}\;\varphi (\Omega ) \end{array}$$

(3b) $$\begin{array}{cc} \rho a-\nabla \cdot \sigma =\rho b\; & \mathrm{in}\;\varphi (\Omega ) \end{array}$$

where ρ is the mass density, a is the acceleration, v is the velocity, σ is the symmetric Cauchy stress tensor and b is the body force. Acceleration and velocity are, by definition, the material derivatives of the velocity, v, and the displacement, u, respectively. For a compressible material, the conservation of mass is satisfied by:

(5) $$\rho =\frac{\rho_{0}}{det(F)}$$

where $\rho_{0}$ is the density in the undeformed configuration and det(F) is the determinant of the total deformation gradient F = dx/dX with x and X representing the current and initial position, respectively. Equation (4) holds at any point and in particular at the sampling points where the equation is written, e.g., the material points. Thermal effects are not considered in the present work, so the energy balance is considered implicitly fulfilled.

The balance equations are solved numerically in a three-dimensional region $\Omega \subseteq R^{3}$, in the time range t∈[0,T], given the following boundary conditions on the Dirichlet ($\varphi (\partial \Omega_{D})$) and Neumann boundaries ($\varphi (\partial \Omega_{N})$), respectively:

(5a) $$\begin{array}{cc} u & =\bar{u}\;\mathrm{on}\;\varphi \left(\partial \Omega_{D}\right) \end{array}$$

(5b) $$\begin{array}{cc} \sigma \cdot n & =\bar{t}\;\mathrm{on}\;\varphi \left(\partial \Omega_{N}\right) \end{array}$$

where n is the unit outward normal.

A constitutive equation for evaluation of the stress-strain relation is also needed to fully define the boundary value problem.

## 3. Weak Form

In Section 2, the strong form of the problem has been defined. In this section, the weak form is derived, following the formulation explained in [48], a displacement-based finite element procedure.

Let the displacement space $V\in {\left[H^{1}\left(B\right)\right]}^{d}$ be the space of vector functions whose components and their first derivatives are square-integrable; the integral form of the problem is:

(6) $$\begin{array}{cc} \int_{\varphi (\Omega )}\left(\nabla \cdot \sigma \right)\cdot wdv+\int_{\varphi (\Omega )}\rho \left(b-a\right)\cdot wdv-\int_{\varphi (\partial \Omega_{N})}\left(\sigma \cdot n-\bar{t}\right) & \cdot wda=0,\\ & \;\forall w\in V \end{array}$$

where w is an arbitrary test function, such that $w=\left\{w\in V|w=0\;\mathrm{on}\;\varphi (\partial \Omega_{D})\right\}$, dv is the differential volume and da the differential boundary surface. By integrating by parts, applying the divergence theorem and considering the symmetry of the stress tensor, the following expression is obtained:

(7) $$\int_{\varphi (\Omega )}\sigma :\left(\nabla^{S}w\right)dv-\int_{\varphi (\Omega )}\rho \left(b-a\right)\cdot wdv-\int_{\varphi (\partial \Omega_{N})}\bar{t}\cdot wda=0$$

Under the assumption that the stress tensor is a function of the current strain only:

(8) σ=σ(ϵ)

the problem is reduced to find a kinematically-admissible field u that satisfies:

(9) G(u,w)=0∀w∈V

where *G* is the virtual work functional defined as:

(10) $$G\left(u,w\right)=\int_{\varphi (\Omega )}\sigma :\left(\nabla^{S}w\right)dv-\int_{\varphi (\Omega )}\rho \left(b-a\right)\cdot wdv-\int_{\varphi (\partial \Omega_{N})}\bar{t}\cdot wda$$

### 3.1. Linearization of the Spatial Weak Formulation

In this work, we attempt to solve the general Boundary Value Problems (BVP), characterized by both geometrical and material non-linearity. When a non-linear BVP is considered, the discretisation of the weak form results in a system of non-linear equations; for the solution of such a system, a linearization is, therefore, needed. The most used and known technique is Newton–Raphson’s iterative procedure, which makes use of directional derivatives to linearize the non-linear equations.

The virtual work functional of Equation (10) is linearized with respect to the unknown u, using an arbitrary argument $u^{*}$, which is chosen to be the last known equilibrium configuration. The linearized problem is to find δu such that:

(11) $$L\left(\delta u,w\right)≃G\left(u^{*},w\right)+DG\left(u^{*},w\right)\left[\delta u\right]=0,\;\forall w\in V$$

where *L* is the linearized virtual work function and:

(12) $$DG\left(u^{*},w\right)\left[\delta u\right]=\frac{d}{d\gamma }{|}_{\gamma =0}G\left(u^{*}+\gamma \delta u,w\right)$$

is the directional derivative of *G* at $u^{*}$ in the direction of δu, given by:

(13) $$\begin{array}{cc} DG\left(u^{*},w\right)\left[\delta u\right] & =\frac{d}{d\gamma }{|}_{\gamma =0}\int_{\varphi (\Omega )}\left[\sigma \left(\epsilon \left(\gamma \right)\right):\left(\nabla^{S}w\right)-\rho \left(b-a\right)\cdot w\right]dv\\ & -\frac{d}{d\gamma }{|}_{\gamma =0}\int_{\varphi (\partial \Omega_{N})}\bar{t}\cdot wda \end{array}$$

Under the assumption of conservative external loads, only the terms related to the internal and inertial forces are dependent on the deformation. Using the following definitions:

(14) $$\epsilon \left(\gamma \right)=\nabla^{S}\left(u^{*}+\gamma \delta u\right)=\epsilon^{*}+\gamma \nabla^{S}\left(\delta u\right)$$

where $\epsilon^{*}=\nabla^{S}\left(u^{*}\right)$ is the strain field at $u^{*}$ and $u\left(\gamma \right)=u^{*}+\gamma \delta u$, the directional derivative $DG\left(u^{*},w\right)\left[\delta u\right]$ reduces to:

(15) $$\begin{array}{cc} DG\left(u^{*},w\right)\left[\delta u\right] & =\frac{d}{d\gamma }{|}_{\gamma =0}\left(\int_{\varphi (\Omega )}\left[\sigma \left(\epsilon \left(\gamma \right)\right):\left(\nabla^{S}w\right)+\rho a\left(u\left(\gamma \right)\right)\cdot w\right]dv\right)\\ & =DG^{static}\left(u^{*},w\right)\left[\delta u\right]+DG^{dynamic}\left(u^{*},w\right)\left[\delta u\right] \end{array}$$

which can be split into a static and dynamic contribution.

Under the assumption of finite strains and adopting an updated Lagrangian kinematic framework, the expression of the directional derivative (Equation (15)) should be derived in spatial form. A common way to do that consists of linearizing the material weak form and in doing a push-forward operation to recover the spatial form [48]. Therefore, the linearization of the weak form derived with respect to the initial configuration reads:

(16) $$\begin{array}{cc} DG\left(u^{*},w\right)\left[\delta u\right] & =\int_{\Omega }\nabla_{X}\delta uS\cdot \nabla_{X}wdV\\ & +\int_{\Omega }\left[\left(F^{T}\nabla_{x}^{S}wF\right):C\left(F^{T}\nabla_{x}^{S}\delta uF\right)\right]dV\\ & +\int_{\Omega }\rho_{0}\frac{da}{du}\cdot w\left[\delta u\right]dV \end{array}$$

where $\nabla_{X}$ and $\nabla_{x}$ are the material and spatial gradient operator, respectively, S is the second Piola–Kirchhoff stress tensor, C is the fourth order incremental constitutive tensor and dV is the differential volume element in the undeformed configuration. The linearization of the weak form with respect to the current configuration can be derived by pushing forward the linearization of Equation (16). The first term can be directly written in terms of the Kirchhoff stress $\tau =FSF^{T}$ as:

(17) $$\nabla_{X}\delta uS\cdot \nabla_{X}w=\nabla_{X}\delta uF^{-1}\tau F^{-T}\cdot \nabla_{X}w$$

and using this standard identity $\nabla_{x}a=\nabla_{X}aF^{-1}$, Equation (17) can be written as:

(18) $$\nabla_{X}\delta uS\cdot \nabla_{X}w=\nabla_{x}\delta u\tau \cdot \nabla_{x}w$$

The second integral of Equation (16) can be re-written as:

(19) $$\int_{\Omega }\nabla_{x}^{S}w:\hat{C}\left[\nabla_{x}^{S}\delta u\right]dV$$

adopting the transformation of the fourth order incremental constitutive tensor C in Voigt notation [48]:

(20) $${\hat{C}}_{iklm}=F_{iA}F_{lC}F_{mD}F_{kB}C_{ABCD}$$

where lowercase indexes refer to the incremental constitutive tensor relative to the Kirchhoff stress, while uppercase indexes to the incremental constitutive tensor relative to the second Piola–Kirchhoff stress.

With these transformations, the linearization of the static contribution at the current configuration is:

(21) $$DG^{static}\left(u^{*},w\right)\left[\delta u\right]=\int_{\Omega }\nabla_{x}\delta u\tau \cdot \nabla_{x}w+\nabla_{x}^{S}w:\hat{C}\left[\nabla_{x}^{S}\delta u\right]dV$$

Considering the definition of the determinant of the deformation gradient:

(22) $$det\left(F\right)=J=\frac{dv}{dV}$$

the following relations hold

(23) $$\sigma =\frac{1}{J}\tau$$

(24) $$\bar{\hat{C}}=\frac{1}{J}\hat{C}$$

where σ and τ are the Cauchy and Kirchhoff stress tensor, respectively, and $\bar{\hat{C}}$ is the incremental constitutive tensor relative to the Cauchy stress. Equation (16) can now be re-written in the current configuration as:

(25) $$DG\left(u^{*},w\right)\left[\delta u\right]=\int_{\varphi (\Omega )}\left(\nabla_{x}\delta u\sigma \cdot \nabla_{x}w+\nabla_{x}^{S}w:\bar{\hat{C}}\left[\nabla_{x}^{S}\delta u\right]+\rho \frac{da}{du}\cdot w\left[\delta u\right]\right)dv$$

Equation (25) represents the linearization of the spatial weak formulation, also known as the updated Lagrangian formulation, since the deformation state $u^{*}$ is continuously updated during the non-linear incremental solution procedure, e.g., Newton–Raphson’s method.

### 3.2. Spatial Discretisation

For the sake of clarity, hereinafter, the *p* subscript is used to refer to variables attributed to the material points, while the *I* subscript is used to refer to variables attributed to the computational nodes. Let us assume discretizing the continuum body B by a set of $n_{p}$ material points and assigning a finite volume of the body $\Omega_{p}$ to each of those material points. Thus, the geometrical representation ($B_{h}$) of B reads:

(26) $$B\approx B_{h}=\overset{n_{p}}{\underset{p=1}{⋃}}\Omega_{p}$$

and the integrals of the weak form can be written as:

(27) $$\int_{B}\left(\ldots \right)dV\approx \int_{B^{h}}\left(\ldots \right)dV=\overset{n_{p}}{\underset{p=1}{⋃}}\int_{\Omega_{p}}\left(\ldots \right)d\Omega_{p}$$

Let $V_{h}$ be a finite element space to approximate V. The problem is now finding $u_{h}\in V_{h}$ such that:

(28) $$DG\left(u_{h}^{*},w_{h}\right)\left[\delta u_{h}\right]=-G\left(u_{h}^{*},w_{h}\right),\;\forall w_{h}\in V_{h}$$

or using Equation (25):

(29) $$\begin{array}{c} \int_{\varphi (\Omega )}\left\{\nabla_{x}\delta u_{h}\sigma \cdot \nabla_{x}w_{h}+\nabla^{S}w_{h}:\bar{\hat{C}}\left[\nabla^{S}\delta u_{h}\right]+\rho \frac{da_{h}}{du_{h}}\cdot w_{h}\left[\delta u_{h}\right]\right\}dv=\\ -\left(\int_{\varphi (\Omega )}\sigma :\left(\nabla^{S}w_{h}\right)dv-\int_{\varphi (\Omega )}\rho \left(b-a_{h}\right)\cdot w_{h}dv-\int_{\varphi (\partial \Omega_{N})}\bar{t}\cdot w_{h}da\right) \end{array}$$

The detailed procedure to obtain the linearization of Equation (29) can be found in [48].

The final discretized form can be written as:

(30) $$\begin{array}{c} \overset{n_{p}}{\underset{p=1}{⋃}}\sum_{I=1}^{n}\sum_{K=1}^{n}w_{I}^{T}\left({\left(\nabla_{x}N_{I}\right)}^{T}\sigma \left(\nabla_{x}N_{K}\right)I+B_{I}^{T}DB_{K}+\frac{N_{I}\rho N_{K}}{\beta \Delta t^{2}}I\right)V_{p}\delta u_{K}\\ =-\overset{n_{p}}{\underset{p=1}{⋃}}\sum_{I=1}^{n}w_{I}^{T}\left(B_{I}\sigma -\rho bN_{I}+\sum_{K=1}^{n}N_{I}\rho N_{K}a_{K}\right)V_{p}-\overset{n_{l}}{\underset{l=1}{⋃}}\sum_{I=1}^{n_{m}}w_{I}^{T}N_{I}\bar{t}A_{l} \end{array}$$

where *I* and *K* are the indexes of the finite element’s nodes, $\nabla_{x}N_{I}$ is the spatial gradient of the shape function evaluated at node *I*, D is the matrix form of the incremental constitutive tensor $\bar{\hat{C}}$, $V_{p}$ is the volume relative to a single material point, $A_{l}$ is the surface and $B_{I}$ is the deformation matrix relative to node *I*, expressed here for a 2D problem as:

(31) $$B_{I}=\left[\begin{array}{cc} \frac{\partial N_{I}}{\partial x} & 0\\ 0 & \frac{\partial N_{I}}{\partial y}\\ \frac{\partial N_{I}}{\partial y} & \frac{\partial N_{I}}{\partial x} \end{array}\right]$$

The left-hand side of Equation (30) is given by three addends multiplied by the increment of the unknowns. The first one is commonly known as the geometric stiffness matrix:

(32) $$K_{IK}^{geo}={\left(\nabla_{x}N_{I}\right)}^{T}\sigma \left(\nabla_{x}N_{K}\right)IV_{p}$$

while the second term is known as the material stiffness matrix:

(33) $$K_{IK}^{mat}=B_{I}^{T}DB_{K}V_{p}$$

and their sum represents the static contribution to the tangent stiffness matrix:

(34) $$K_{IK}^{static}=K_{IK}^{geo}+K_{IK}^{mat}$$

The dynamic component is given by:

(35) $$K_{IK}^{dynamic}=\frac{N_{I}\rho N_{K}}{\beta \Delta t^{2}}IV_{p}$$

and its definition depends on the adopted time scheme as explained in Section 4.

Finally, the tangent stiffness matrix is given by:

(36) $$K_{IK}^{tan}=K_{IK}^{static}+K_{IK}^{dynamic}$$

and represents the submatrix relative to one node of the discretisation with dimension $\left[n_{dof}\times n_{dof}\right]$, where $n_{dof}$ is the number of degrees of freedom of a single node. This matrix can be considered as the Jacobian matrix of the right-hand side of Equation (30), i.e., the residual $R_{I}$. Equation (30) can be rewritten in compact form as:

(37) $$K_{IK}^{tan}\delta u_{K}=-R_{I}.$$

## 4. MPM and GMM Techniques

In Section 3, the formulation, common to both techniques, based on an updated Lagrangian description of the continuum, is presented. In this section, the characteristic featrues of MPM and GMM are highlighted.

As previously stated, MPM and GMM are two Lagrangian techniques and share the fact that the material points shall be understood as the integration points of the calculation, each carrying information about the material and kinematic response. Each material point represents a computational element with one single integration point (the material point itself), whose connectivity is defined by either the nodes of the elements in which it falls (in MPM) or by the nodes in the cloud around the material point (in GMM). In both algorithms, the initial position of the material points is chosen to coincide with the Gauss points of an FE grid, and the mass, which remains constant during the simulation, is equally distributed between the material points, falling, initially, in the same element.

**Figure 1:**
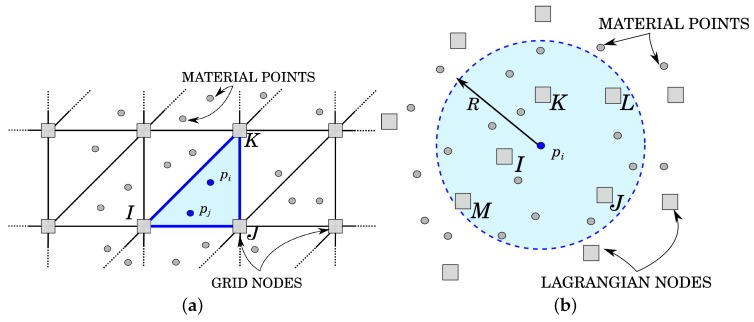
Shape functions calculation in MPM and Galerkin Meshfree Method (GMM). (**a**) MPM. The shape functions on the material point $p_{i}$ are evaluated using the FE shape function of element I-J-K; (**b**) GMM. The shape functions on the material point $p_{i}$ are evaluated using the information on the nodes sufficiently close to the material point itself.

In MPM, such material points may migrate from one element of the grid to another. In the evaluation of the FEM integrals, the shape functions are evaluated at the material point location on the basis of the grid element into which the material point falls (Figure 1a). To prevent mesh distortion, the nodal solution is deleted such that at each time step, the undeformed mesh is recovered; while in GMM, the material points move together with the computational nodes, and the shape functions are evaluated once the surrounding cloud of nodes is defined (Figure 1b). In this case the nodes preserve their history through the whole simulation, as in FEM.

### 4.1. MPM

MPM is a particle method, proposed for the first time by Harlow [49] for the solution of fluid flow problems under a large deformation regime and originally known by the name of the Particle-In-Cell (PIC) method. Some decades after, Sulsky and coworkers presented its extension to solid mechanics problems [35,36]. As previously stated, most MPM codes make use of explicit time schemes, which are generally preferable when simulating impacts at high velocities or fast transient problems. In other cases, for example when the driving force is gravity or when the rate of deformation is small, the adoption of an implicit time scheme is the best choice, since the stability of the method (for properly chosen dissipative methods) does not depend on the wave propagation speed within the media, which provides the typical time step limitation for explicit approaches [50].

In the current work, the displacement-based formulation and the time scheme integration of the MPM algorithm are equivalent to those proposed by [41].

#### 4.1.1. MPM Algorithm

**Figure 2:**
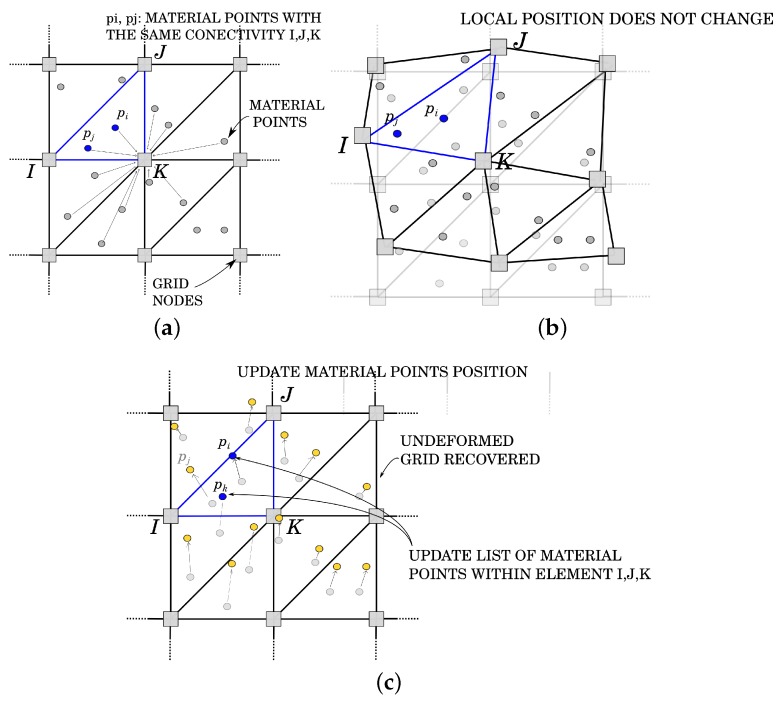
MPM phases. (**a**) Initialization phase; (**b**) Updated Lagrangian FEM phase; (**c**) Convective phase.

Traditionally, the MPM algorithm is composed of three different phases [36], as graphically represented in Figure 2:

(a) Initialization phase(Figure 2a): At the beginning of the time step, the connectivity is defined for each material points, and the initial conditions on the FE grid nodes are created by means of a projection of material points’ information obtained at the previous time step $t_{n}$;
(b) UL-FEM calculation phase (Figure 2b): The local matrix, represented by the left-hand-side (lhs) of Equation (30), and the local vector, constituted by the right-hand-side (rhs) of Equation (30), are evaluated in the current configuration according to the formulation presented in the previous section. The global left-hand-side matrix (LHS) and the global right-hand-side vector (RHS) are obtained by assembling the local contributions of each material point, and finally, the system is iteratively solved. During the iterative procedure, the nodes are allowed to move, according to the nodal solution, and the material points do not change their local position within the geometrical element until the solution has reached convergence;
(c) Convective phase (Figure 2c): During the third and last phase, the nodal information at time $t_{n+1}$ is interpolated back to the material points. The position of the material points is updated and, in order to prevent mesh distortion, the undeformed FE grid is recovered.

Many featrues of the MPM are connected to the finite element method [35]. Indeed, Phase b coincides with the calculation step of a standard non-linear FE code, while Phases a and c define the MPM featrues.

At the beginning of each time step ($t_{n}$), during Phase a, the degrees of freedom and the variables on the nodes of the fixed mesh are defined gathering the information from the material points (Figure 2a).

The momentum $q_{p}$ and inertia $f_{p}$ on the material points, which are defined as functions of mass $m_{p}$, velocity $v_{p}$ and acceleration $a_{p}$:

(38) $$q_{p}^{n}=v_{p}^{n}m_{p}$$

(39) $$f_{p}^{n}=a_{p}^{n}m_{p}$$

are projected on the background grid by evaluating in a first step the global values of nodal mass $m_{I}$, momentum $q_{I}$ and inertia $f_{I}$ as described in Algorithm 1.

Once $m_{I}$, $q_{I}$ and $f_{I}$ are obtained, it is possible to compute the values of nodal velocity and nodal acceleration of the previous time step as:

(40) $${\tilde{v}}_{I}^{n}=\frac{q_{I}^{n}}{m_{I}}$$

(41) $${\tilde{a}}_{I}^{n}=\frac{f_{I}^{n}}{m_{I}}$$

It is worth mentioning that the initial nodal conditions are evaluated at each time step using material point information in order to have initial values even on grid elements empty at the previous time step ($t_{n-1}-t_{n}$).

Both Lagrangian techniques presented in this paper make use of a predictor/corrector procedure, based on the Newmark integration scheme.

In MPM, the prediction of the nodal displacement, velocity and acceleration reads:

(42) $${}^{it+1}\Delta u_{I}^{n+1}=0.0$$

(43) $${}^{it+1}v_{I}^{n+1}=\frac{\lambda }{\zeta \Delta t}\left[{}^{it+1}\Delta u_{I}^{n+1}\right]-\left(\frac{\lambda }{\zeta }-1\right){\tilde{v}}_{I}^{n}-\frac{\Delta t}{2}\left(\frac{\lambda }{\zeta }-2\right){\tilde{a}}_{I}^{n}$$

(44) $${}^{it+1}a_{I}^{n+1}=\frac{1}{\zeta \Delta t^{2}}\left[{}^{it+1}\Delta u_{I}^{n+1}\right]-\frac{1}{\zeta \Delta t}{\tilde{v}}_{I}^{n}-\left(\frac{1}{2\zeta }-1\right){\tilde{a}}_{I}^{n}$$

where the upper-left side index it indicates the iteration counter, while the upper-right index *n* the time step. λ and ζ are Newmark’s coefficients equal to 0.5 and 0.25, respectively.

Once the nodal velocity and acceleration are predicted (Equations (42)–(44)), the system of linearized governing equations is formulated, according to Section 3, and the local matrix $K^{tan}$ and the residual $R_{I}$ are evaluated and assembled according to Equations (30) and (37), respectively (Phase b, Figure 2b).

The solution in terms of increment of nodal displacement is found iteratively solving the residual-based system of Equation (37). Once the solution ${}^{it+1}\delta u_{I}^{n+1}$ is obtained, a correction of the nodal increment of displacement is performed:

(45) $${}^{it+1}\Delta u_{I}^{n+1}{=}^{it}\Delta u_{I}^{n+1}{+}^{it+1}\delta u_{I}^{n+1}$$

Velocity and acceleration are corrected according to Equations (43) and (44), respectively. This procedure has to be repeated until convergence is reached.

Unlike an FEM code, the nodal information is available only during the calculation of a time step: at the beginning of each time step, a reset of all the nodal information is performed, and the accumulated displacement information is deleted. The computational mesh is allowed to deform only during the iterative procedure of a time step, avoiding the typical element tangling of a standard FEM. When convergence is achieved, the position of the nodes is restored to the original one (Phase c, Figure 2c). Before restoring the undeformed configuration of the FE grid, the solution in terms of nodal displacement, velocity and acceleration is interpolated on the material points, as:

(46) $$\Delta u_{p}^{n+1}=\sum_{n=1}^{n_{n}}N_{I}\left(\xi_{p},\eta_{p}\right)\Delta u_{I}^{n+1}$$

(47) $$a_{p}^{n+1}=\sum_{n=1}^{n_{n}}N_{I}\left(\xi_{p},\eta_{p}\right)a_{I}^{n+1}$$

(49) $$v_{p}^{n+1}=v_{p}^{n}+\frac{1}{2}\Delta t\left(a_{p}^{n}+a_{p}^{n+1}\right)$$

where $n_{n}$ is the total number of nodes per geometrical element, $\left(\xi_{p},\eta_{p}\right)$ are the local coordinates of material point *p* and $N_{I}\left(\xi_{p},\eta_{p}\right)$ is the shape function evaluated at the position of the material point *p*, relative to node *I*.

Finally, the current position of the material points is updated as:

(49) $$x_{p}^{n+1}=x_{p}^{n}+\Delta u_{p}^{n+1}$$

The details of the MPM algorithm are presented in Algorithm 1.

**Algorithm 1** MPM algorithm.(we will use ${\left(•\right)}^{n}=\left(•\right)\left(t_{n}\right)$)Material DATA: E, ν, ρInitial data on material points: $m_{p}$, $x_{p}^{n}$, Δt, $u_{p}^{n},v_{p}^{n},a_{p}^{n},F_{p}^{n}=\sum_{I}\frac{\partial N_{I}}{\partial x_{I}^{0}}\cdot x_{I}^{n}$
$\Delta F_{p}=\sum_{I}\frac{\partial N_{I}}{\partial x_{I}^{n}}\cdot x_{I}^{n+1}$Initial data on nodes: **NONE - everything is discarded in the initialization phase**OUTPUT of calculations: $\Delta u_{I}^{n+1},\sigma_{p}^{n+1}$**INITIALIZATION PHASE**
Clear nodal info and recover undeformed grid configurationCalculation of initial nodal conditions.
(a)for p = 1:$N_{p}$
*Calculation of nodal data
·$q_{I}^{n}=\sum_{p}N_{I}\;m_{p}v_{p}^{n}$·$f_{I}^{n}=\sum_{p}N_{I}m_{p}a_{p}^{n}$·$m_{I}^{n}=\sum_{p}N_{I}m_{p}$

(b)for I = 1:$N_{I}$
*${\tilde{v}}_{I}^{n}=\frac{q_{I}^{n}}{m_{I}^{n}}$*${\tilde{a}}_{I}^{n}=\frac{f_{I}^{n}}{m_{I}^{n}}$

Newmark method: PREDICTOR. Evaluation of ${}^{it+1}\Delta u_{I}^{n+1}$, ${}^{it+1}v_{I}^{n+1}$ and ${}^{it+1}a_{I}^{n+1}$ using Equations (42)–(44)
**UL-FEM PHASE**
for p = 1:$N_{p}$
(a)Evaluation of local residual (rhs) (Equation (10))(b)Evaluation of local Jacobian matrix of residual (lhs) (Equation (25))(c)Assemble rhs and lhs to the global vector RHS and global matrix LHS (Equations (30) and (37))
Solving system $\left(\Delta u_{I}^{n+1}\right)$Newmark method: CORRECTOR (Equations (43)–(45))Check convergence
(a)NOT converged: go to Step 2(b)Converged: go to Step 3

**CONVECTIVE PHASE**
Update the kinematics on the material points by means of an interpolation of nodal information (Equations (46)–(49))Save the stress $\sigma_{p}^{n+1}$, strain $\epsilon_{p}^{n+1}$ and total deformation gradient $F_{p}^{n+1}$ on material points (the latter by $F_{p}^{n+1}=\Delta F_{p}\cdot F_{p}^{n}$)


### 4.2. GMM

The GMM is a truly meshless method that can be seen as the application of the MPM idea extended to the case in which both the nodes and the material points behave as purely Lagrangian through the whole analysis. Thus, it is relatively easy to enforce conservation properties at the integration points, while also maintaining the history of nodal results during all the simulation time, provided that a reliable technique is chosen for the computation of the meshless shape functions. The difficulty is, hence, moved to the construction of such an effective meshless base, which is addressed in Section 4.2.2.

#### 4.2.1. GMM Algorithm

**Figure 3:**
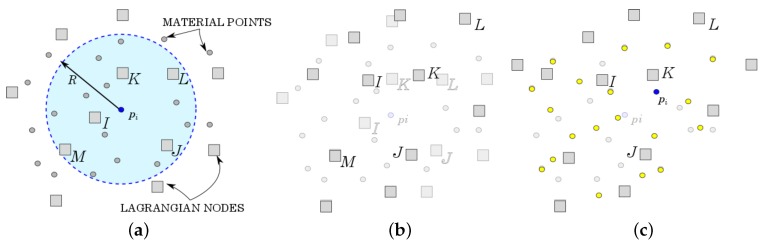
GMM phases. (**a**) Initialization phase; (**b**) Updated Lagrangian FEM phase; (**c**) Convective phase.

The GMM algorithm is based on three principal steps (see Figure 3). The initialization phase (Figure 3a) is the step that mostly distinguishes GMM from MPM. During this phase, the connectivity of each integration point (i.e., each material point) is computed as the “cloud of nodes”, centered on the material point and obtained by a search-in-radius. Such a cloud is then employed for the calculation of the shape functions. Unlike MPM, the Newmark prediction is performed by using the nodal information of the previous time step, like in FEM. Once *N* and ∇N are suitably defined, MPM and GMM essentially coincide in the following steps. This is reflected in Steps 2 (Figure 3b) and 3 (Figure 3c) of the algorithm being coincident with the MPM case. The details of the GMM algorithm are presented in Algorithm 2.

**Algorithm 2** GMM algorithm.Material DATA: E, ν, ρInitial data on material points: $m_{p}$, $x_{p}^{n}$, $\Delta t,F_{p}^{n}=\sum_{I}\frac{\partial N_{I}}{\partial x_{I}^{0}}\cdot x_{I}^{n}$$\Delta F_{p}=\sum_{I}\frac{\partial N_{I}}{\partial x_{I}^{n}}\cdot x_{I}^{n+1}$Initial data on nodes: $u_{I}^{n},v_{I}^{n},a_{I}^{n}$OUTPUT of calculations: $u_{I}^{n+1}\sigma_{p}^{n+1}$**INITIALIZATION PHASE**
for every material point with position $x_{p}^{n}$ gather the cloud of nodes with position $x_{I}^{n}$ such that $\left|x_{p}-x_{I}\right|<R$compute the shape functions $N_{I}\left(x_{p}^{n}\right)$ for all nodes *I* in the cloudNewmark method: PREDICTORfor the prediction of displacement, unlike Equation (42), ${}^{it+1}u_{I}^{n+1}=u_{I}^{n}$,while for the prediction of nodal velocity and nodal acceleration, see Equations (43) and (44)
**UL-FEM PHASE** (identical to MPM)**CONVECTIVE PHASE**
Update position of the material points by means of an interpolation of nodal solution
(a)for p = 1:$N_{p}$$x_{p}^{n+1}=x_{p}^{n}+\sum N_{I}\Delta u_{I}^{n+1}$
Save state of stress $\sigma_{p}^{n+1}$, state of strain $\epsilon_{p}^{n+1}$ and total deformation gradient $F_{p}^{n+1}$ on material points (the latter by $F_{p}^{n+1}=\Delta F_{p}\cdot F_{p}^{n}$)



#### 4.2.2. Calculation of GMM Shape Functions

While the computation of the shape functions is trivial for the standard MPM, thanks to the presence of a background grid (Figure 1a), the evaluation of the shape functions in GMM is more complex. From a technical point of view, GMM is based on a conceptually simple operation: given an arbitrary position $x_{p}$ in space (which will, in practice, coincide with the position of the material point) and a search radius *R*, one may find all of the nodes *I* such that $||x_{I}-x_{p}||<R$. Given such a cloud of nodes, one may then compute, at the position $x_{p}$, the shape functions $N_{I}\left(x_{p}\right)$ (together with their gradients), such that, a given function *u*, whose nodal value is $u_{I}$, can be interpolated at the position $x_{p}$ as $u\left(x_{p}\right)=\sum_{I}N_{I}\left(x_{p}\right)\;u_{I}$ (Figure 1b).

However, in order to construct a convergent solution, some guarantees must be provided by the shape functions. In particular, they shall comply with the Partition of Unity (PU) property, as a very minimum at all of the positions $x_{p}$ at which the shape functions are evaluated. A number of shape functions exist complying with such a property [51,52,53]. Among the available options, two appealing classes of meshless functions are considered in our work: the first choice is constituted by the so-called Moving Least Square (MLS) method and the second one represented by the Local Maximum Entropy (LME) technique.

The first technique is based on the MLS approach, first introduced by Lancaster [54] and Belytschko [44,52]. The MLS-approximation fulfills the reproducing conditions by construction, so no corrections are needed.

The fundamental principle of MLS approximants is based on a weighted least square fitting of a target solution, sampled at a given, possibly randomly-distributed, set of points, via a function of the type:

(50) $$P\left(x\right)=a_{1}+a_{2}x+a_{3}y+a_{4}xy+\cdots$$

where the coordinates x,y are to be understood as relative to the sampling position.

The reconstruction of a continuous function h(x) can be obtained considering the data $h_{I}$ be located at points $x_{I}$ and an arbitrary, smooth and compactly supported, weight function $W_{I}\left(x\right)$, such that the $x_{I}$ fall within the support of *W*. Assuming now that the reconstructed function ($h_{x}^{h}$) is computed as:

(51) $$h\left(x\right)\approx h_{x}^{h}=P^{T}\left(x\right)\cdot a\left(x\right)$$

the fitting to h(x) is done by minimizing the error function *J*, defined as:

(52) $$J=\sum_{I}{\left(P_{I}^{T}\cdot a\left(x\right)-h_{I}\right)}^{2}W_{I}\left(x\right)$$

where $P_{I}=P\left(x_{I}\right)$.

This allows defining a set of approximating shape functions *N* such that:

(53) $$h\left(x\right)=\sum_{I}N_{I}\left(x\right)h_{I}$$

where:

(54) $$N_{I}=P^{T}\left(x\right)\cdot M^{-1}\left(x\right)\cdot P_{I}W_{I}\left(x\right)$$

with M defined as:

(55) $$M\left(x\right)=\sum_{I}P_{I}P_{I}^{T}W_{I}\left(x\right)$$

It can be readily verified that the shape functions are able to reproduce exactly a polynomial up to the order used in the construction. This fact can also be used to prove compliance with the partition of unity property. Namely, if one assumes $h_{I}=P_{I}\left(x_{I}\right)$ and substitutes into Equation (53), then:

(56) $$\begin{array}{cc} \sum_{I}N_{I}\left(x\right)P_{I}^{T} & =P^{T}\left(x\right)\cdot M^{-1}\left(x\right)\cdot \sum_{I}P_{I}P_{I}^{T}W_{I}\left(x\right)\\ & =P^{T}\left(x\right)\cdot M^{-1}\left(x\right)\cdot M\left(x\right)\\ & =P^{T}\left(x\right) \end{array}$$

Hence, considering the special case of a constant polynomial P(x)=1 or of a linear variation in x, P(x)=x we obtain, respectively:

(57) $$\begin{array}{c} \sum_{I}N_{I}\equiv 1\\ \sum_{I}x_{I}N_{I}\equiv x \end{array}$$

A similar reasoning also gives:

(58) $$\begin{array}{c} \sum_{I}\nabla N_{I}\equiv 0\\ \sum_{I}x_{I}\cdot \nabla N_{I}\equiv 1 \end{array}$$

thus proving the compliance with the PU property.

However, MLS shape functions are not able to guarantee the Kronecker-delta property at the nodes. This implies that two nodal shape functions may be simultaneously non-zero at a given nodal position. This has practical implications at the moment of imposing Dirichlet boundary conditions, namely in order to impose $u(x_{d})=0$ at a given point on the Dirichlet boundary $x_{d}$, one must impose that $\sum (N_{I}\left(x_{d}\right)u_{I})=0$, which constitutes a classical multipoint constraint [55].

Interestingly, the choice of different shape functions could ease this particular problem. An appealing choice could be the use of LME approximants, which guarantee complying with a weak Kronecker-delta property until the cloud of nodes is represented by a convex hull.

The LME technique is based on the evaluation of the local max-ent approximants [56], which represents the solution that exhibits a (Pareto) compromise between competing objectives: the principle of max-ent [57] subject to the constraints:

(59) $$\begin{array}{c} \mathrm{For}\;\mathrm{fixed}\;x\;\mathrm{maximize}\;H\left(N_{1},N_{2},\ldots ,N_{m}\right)=-\sum_{I}N_{I}\ln N_{I},\\ \mathrm{subject}\;\mathrm{to}\;N_{I}\geq 0,\;I=1,\ldots ,n_{node},\\ \sum_{I}N_{I}=1,\;\sum_{I}N_{I}x_{I}=x \end{array}$$

and the objective function interpreted as a measure of locality of the shape functions of the Delaunay triangulation:

(60) $$\begin{array}{c} \mathrm{For}\;\mathrm{fixed}\;x\;\mathrm{minimize}\;U\left(x,N_{1},N_{2},\ldots ,N_{m}\right)=-\sum_{I}N_{I}{\left|x-x_{I}\right|}^{2},\\ \mathrm{subject}\;\mathrm{to}\;N_{I}\geq 0,\;I=1,\ldots ,n_{node},\\ \sum_{I}N_{I}=1,\;\sum_{I}N_{I}x_{I}=x \end{array}$$

The solution to the problem can be found minimizing $\beta U\left(x,N_{1},N_{2},\ldots ,N_{m}\right)-H\left(N_{1},N_{2},\ldots ,N_{m}\right)$ subjected to the usual constrains. The optimization problem takes the form:

(61) $$\begin{array}{c} \mathrm{For}\;\mathrm{fixed}\;x\in \mathrm{conv}X,\;\mathrm{minimize}\;\sum_{I}\beta_{I}N_{I}{\left|x-x_{I}\right|}^{2}+\sum_{I}N_{I}\ln N_{I},\\ \mathrm{subject}\;\mathrm{to}\;N_{I}\geq 0,\;I=1,\ldots ,n_{node},\\ \sum_{I}N_{I}=1,\;\sum_{I}N_{I}x_{I}=x \end{array}$$

with $\beta =\gamma /h^{2}$ representing a non-negative locality coefficient, where γ is a dimensionless parameter and *h* is a measure of nodal spacing. The value of γ is always chosen in a range between 0.6, relative to spread-out meshfree shape functions, and four, relative to linear finite element basis functions. Unlike MLS approximants, the LME basis functions possess the weak Kronecker-delta property at the boundary of the convex hull of the nodes, and they are a $C^{\infty }$ function of β in (0,+∞). However, the computation of the LME approximation scheme is more onerous than MLS basis functions, as the problem described by Equation (61) is a convex problem. In Section 5, a comparison between these two procedures is performed through some benchmark tests, and an assessment in terms of computational cost, accuracy and robustness is provided.

## 5. Numerical Examples

In this section, three benchmark tests are considered for the comparison of the MPM and GMM formulations. Firstly, the static analysis of a 2D cantilever beam subjected to its self-weight is analyzed, and a mesh convergence study is performed. Secondly, the rolling of a rigid disk on an inclined plane is studied. Finally, a cohesive-soil column collapse is analyzed. All the numerical experiments have been performed on a PC with one Intel(R) Core(TM) i7-4790 CPU at 3.60 GHz.

### 5.1. 2D Cantilever Beam: Static Analysis

**Figure 4:**
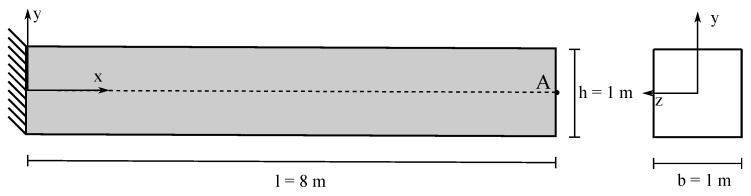
Static 2D cantilever analysis: geometry.

The static analysis of a 2D cantilever beam subjected to its self-weight under the assumption of plain strain is presented. The cantilever beam has a length l=8 m and a square cross-section of unit side (b=h=1 m) (Figure 4). The beam is modeled with a hyperelastic material: the density is ρ=1000 kg/m^3^; the Young’s modulus is E=90 MPa; and the Poisson’s ratio is ν=0. The results obtained with the MPM and GMM algorithms are compared with a standard FEM code using the same UL formulation.

A mesh convergence study is carried out adopting five different mesh sizes, *h* = 0.5, 0.25, 0.125, 0.0625 and 0.01 m, respectively. Quadrilateral elements are used in FEM, MPM and GMM with four integration points per cell (in the case of MPM and GMM, the integration points coincide with the material points). In GMM, the mesh is only initially used for the creation of the material points and then deleted. Regarding the spatial search and the evaluation of the shape functions in GMM, a search radius $R=\sqrt{2h^{2}}$, dilation parameters $R_{eff}=R/2$ and γ=1.8 are adopted in GMM-MLS and GMM-LME, respectively. Under the assumption of a linear regime, the vertical deflection at Point A of the free edge can be evaluated analytically according to Timoshenko [58] as:

(62) $$\delta =-\left(\frac{\rho g\left(bhl\right)l^{3}}{8EI}+\frac{\rho gl^{2}}{2GA_{s}}\right)=-0.67806m$$

where *g* is the gravity acceleration, $I=\frac{bh^{3}}{12}$ the inertia of the beam section and $A_{s}=\frac{5}{6}A$ the reduced cross-section area due to the shear effect. However, the solution is computed under the assumption of non-linearity, and as a benchmark solution, the deflection evaluated through the finest mesh is considered. This value is δ=−0.67433 m and is equally reached by all the methods.

**Figure 5:**
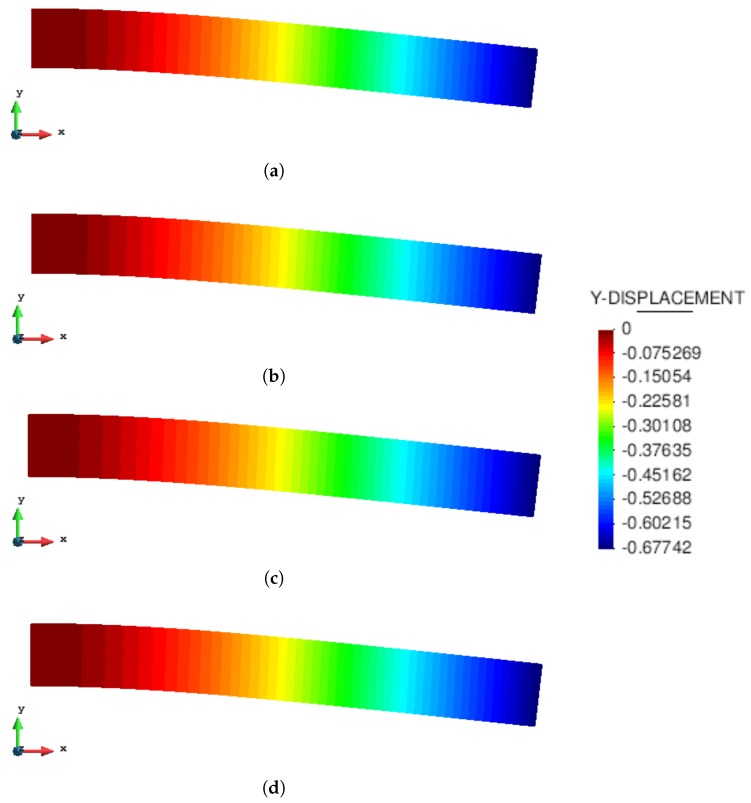
Static cantilever. Displacement along the y-direction.(**a**) FEM code; (**b**) MPM code; (**c**) GMM-MLS code; (**d**) GMM-LME code.

**Figure 6:**
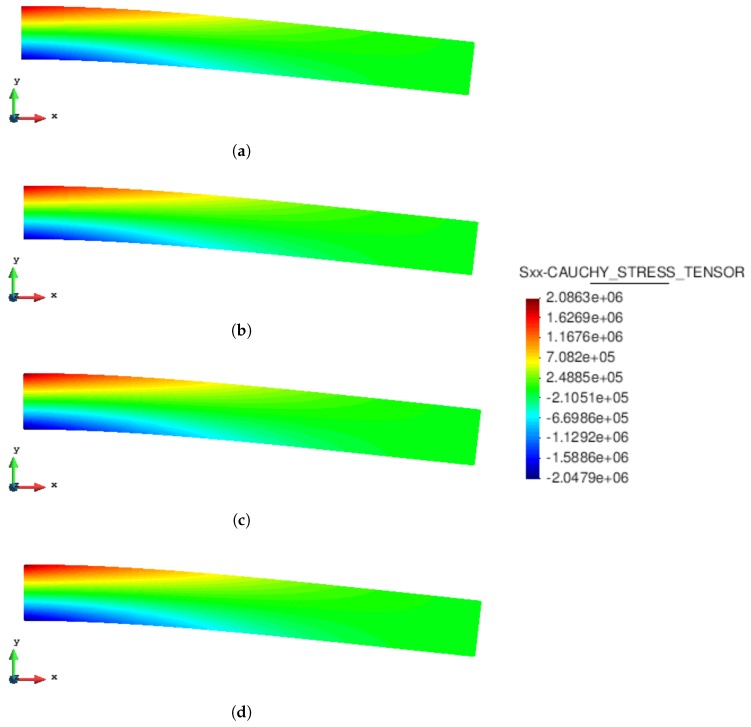
Static cantilever. Cauchy stress along the x-direction. (**a**) FEM code; (**b**) MPM code; (**c**) GMM-MLS code; (**d**) GMM-LME code.

Figure 5 and Figure 6 compare the solutions obtained with an updated Lagrangian FEM, MPM, GMM-MLS and GMM-LME code, respectively, in terms of vertical displacement and Cauchy stress along the horizontal direction. One can observe that the results are in good agreement for all the methods.

**Figure 7:**
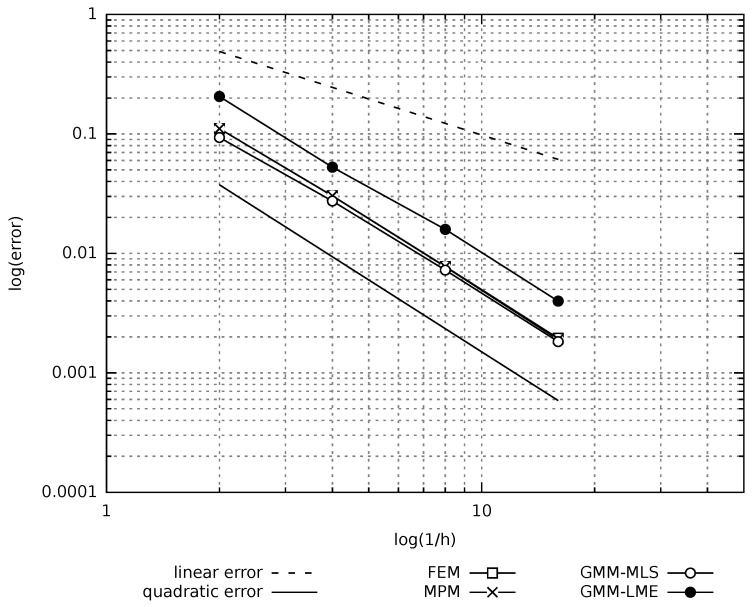
Static cantilever. Convergence analysis.

A convergence study is performed to analyse the accuracy of MPM and GMM in comparison with the UL-FEM. The error is evaluated as:

(63) $$error=\left|\frac{\delta -u_{num}}{\delta }\right|$$

where $u_{num}$ is the numerical solution measure at Point A (see Figure 4). Figure 7 depicts the error evolution as a function of the inverse of the mesh size *h*. It is demonstrated that all the methods have a quadratic rate of convergence. In particular, the UL-FEM, MPM and GMM-MLS error curves coincide. Regarding the error, evaluated with the GMM-LME algorithm, the quadratic rate is maintained, but the curve is shifted a bit upwards, which makes this technique less accurate than GMM-MLS in the benchmark case studied.

### 5.2. Rolling of a Rigid Disk on an Inclined Plane

**Figure 8:**
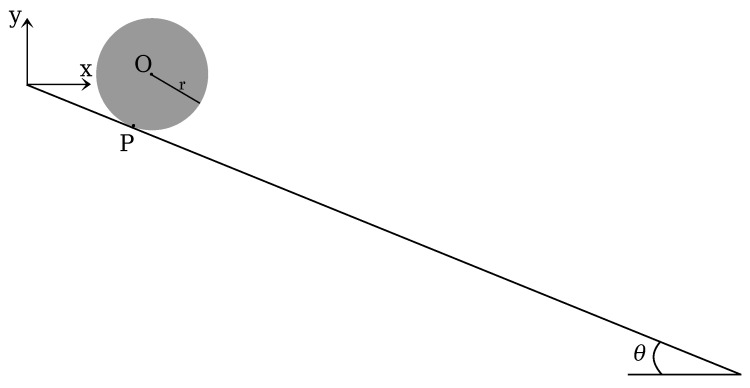
Rolling disk. Geometry.

The second benchmark test is a rigid disk rolling without slipping on an inclined plane. The geometry of the problem is depicted in Figure 8. The disk is made of a hyperelastic material: the density is ρ=7800 kg/m^3^; the Young’s modulus is E=200 MPa; and the Poisson’s ratio is ν=0.3. 

This test is chosen for an objective assessment of the robustness of the MPM and GMM algorithm. The rolling on the plane implies a contact between the nodes belonging to the inclined plane and the nodes belonging to the disk. In a UL-FEM code, a contact algorithm would be necessary to set this boundary condition. On the contrary, by using either MPM or GMM, the contact is implicitly caught. The analytical acceleration (*a*) can be computed imposing the equilibrium of momentum at the contact point *P*:

(64) $$a=\frac{2}{3}g\sin\;\theta$$

where *g* is gravity and θ the angle of the inclined plane. Integrating over time, the acceleration, velocity and displacement projected on the x-axis can be obtained as a function of time:

(65) v(t)=a(t)cosθt

(66) $$u\left(t\right)=v\left(t\right)\cos\;\theta \;t+\frac{1}{2}a\left(t\right)\cos\;\theta \;t^{2}$$

For the study of this test case, the analytical solution of Equation (66) is used for the assessment of the absolute error obtained with MPM, GMM-MLS and GMM-LME, evaluated as:

(67) $$error=\sqrt{\sum_{t_{i}}{\left(u\left(t_{i}\right)-u_{num}\left(t_{i}\right)\right)}^{2}}$$

where $t_{i}$ is the time where the numerical result is calculated. As mesh-based and meshless techniques are compared in a dynamic test, for a more objective comparison, the error is analyzed along with the total computational time needed to finalize the simulation.

A triangular mesh with mesh size h=0.01 m is used for MPM and GMM simulations. In both techniques, the same initial distribution of material points is used, which counts for three initial particles for the cell. Regarding the GMM-MLS the approximants are constructed by adopting a search radius $R=1.5\sqrt{2h^{2}}$ and a dilation parameter $R_{eff}=\sqrt{2h^{2}}$. In GMM-LME, the basis functions are evaluated using a search radius $R=\sqrt{2h^{2}}$ and three values of dilation parameter γ=0.8,1.8,2.8. All the numerical tests are repeated for three different time steps with $\Delta t_{1}=2\Delta t_{2}=4\Delta t_{3}$.

Table 1 shows the results of the analysis, in terms of errors and computational times, performed through MPM, GMM-MLS and GMM-LME.

Regarding the absolute errors, it can be observed that, for a given computational cost, GMM is generally more accurate than MPM, because of the use of smooth basis functions, which provide a better approximation of the unknown variables. In particular, GMM-LME presents smaller errors in comparison to GMM-MLS. In all three cases considered (with γ=0.8,1.8,2.8), the errors converge to a unique value at the same computational time, while in the case of GMM-MLS, the advantage of using higher order elements is lost for the smallest delta time. Regarding MPM, it is established that to achieve the same order of accuracy of GMM, a higher computational time must be expected, due to either a finer discretisation in space or in time. However, it is worth highlighting that GMM is much more time consuming than MPM, showing an increment of computational time of 10% in the case of GMM-MLS and from 8.5% up to 16% in the case of GMM-LME.

In this example, some essential conclusions can be drawn. In Section 5.1, it was observed that in a static case, the rate of convergence is the same for all the methods under analysis, but the accuracy of GMM-MLS and MPM is better than that of GMM-LME. On the contrary, in a dynamic case with a contact problem, the result is overturned. In fact, a better behavior is noted if LME approximants are employed.

### 5.3. Cohesive Soil Column Collapse

The third example is the simulation of a soil column collapse. The column is modeled with a cohesive-frictional material, defined by a cohesion c=5 kPa, a friction angle $\phi =25^{\circ }$, an elastic bulk modulus K=1.5 MPa and a density ρ=1850 kg/m^3^. In the current work, a Mohr–Coulomb plastic law in finite strains is considered, and the implicit integration scheme in principal stress space, presented in [59,60], is followed for its implementation.

This test has been chosen for the assessment of the robustness of MPM and GMM when the body undergoes really large deformation. The results are compared with the work of [31], where a Smooth Particle Hydrodynamics method (SPH) is applied to geotechnical problems.

**Figure 9:**
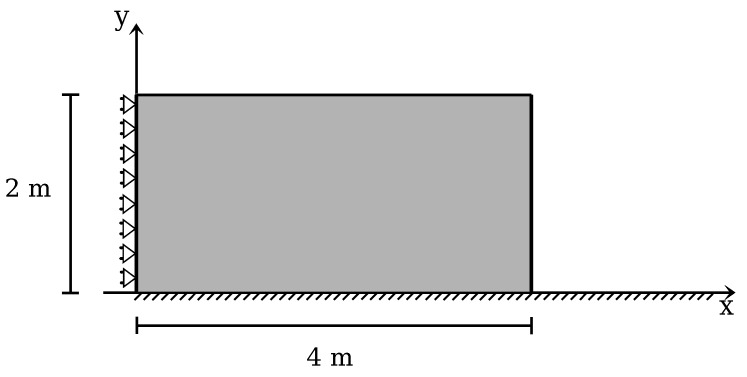
Granular column collapse. Geometry.

The initial geometry and the boundary conditions are described by Figure 9.

Quadrilateral elements with an initial distribution of four material points per cell are used in the simulations. Two different mesh sizes are considered: $h_{1}=0.05$ m (Mesh 1) and $h_{2}=0.025$ m (Mesh 2). In GMM, the basis functions are evaluated using an initial search radius $R=\sqrt{2h^{2}}$, a dilation parameter $R_{eff}=0.5\sqrt{2h^{2}}$ and γ=1.8, in GMM-MLS and GMM-LME, respectively. In this particular case, the procedure for the evaluation of the basis functions in the MLS and LME techniques has been modified to avoid the creation of a non-convex hull of nodes, which might lead to an incorrect set of approximants. This is required because the column is subjected to extremely large deformations. While in the previous examples, a constant radius was used for the definition of the cloud of nodes surrounding a material point, in the current example, a variable radius is adopted to guarantee a minimum number of nodes in each connectivity. In the case of LME, as a Newton iterative procedure is used for the evaluation of the shape functions, a measure of the goodness of the solution is represented by the condition number k(A) of the Hessian matrix *A*, defined in [56]. If k(A) exceeds a user-defined tolerance, the LME algorithm is repeated considering the old connectivity plus an additional node, chosen as the next node closer to the material point. In the case of MLS, it has been sufficient to impose a minimum number of six nodes in each cloud of nodes.

**Figure 10:**
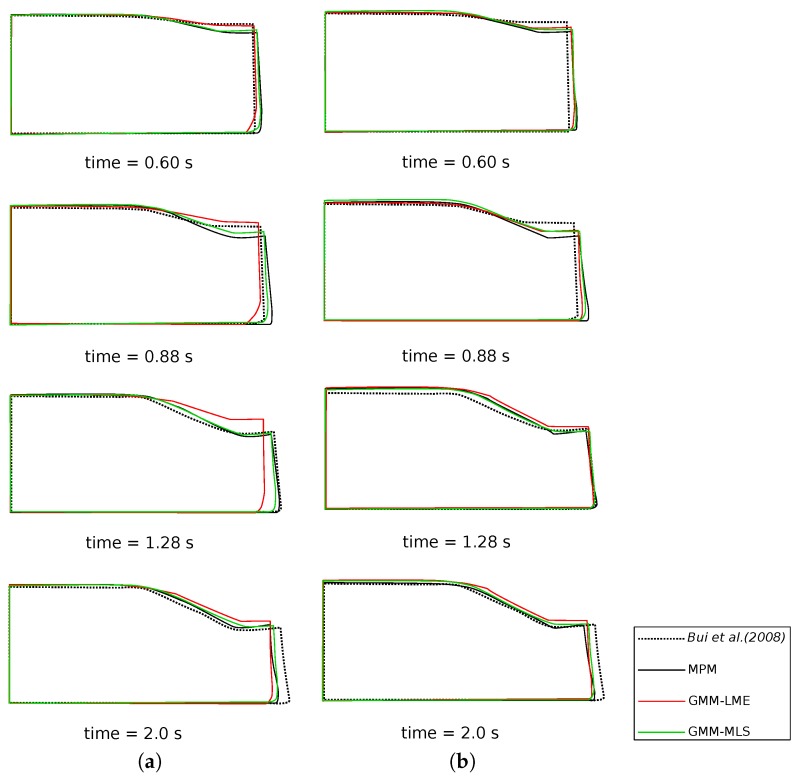
Soil column collapse. Configurations of the column at different representative time instants. (**a**) Mesh 1; (**b**) Mesh 2.

**Figure 11:**
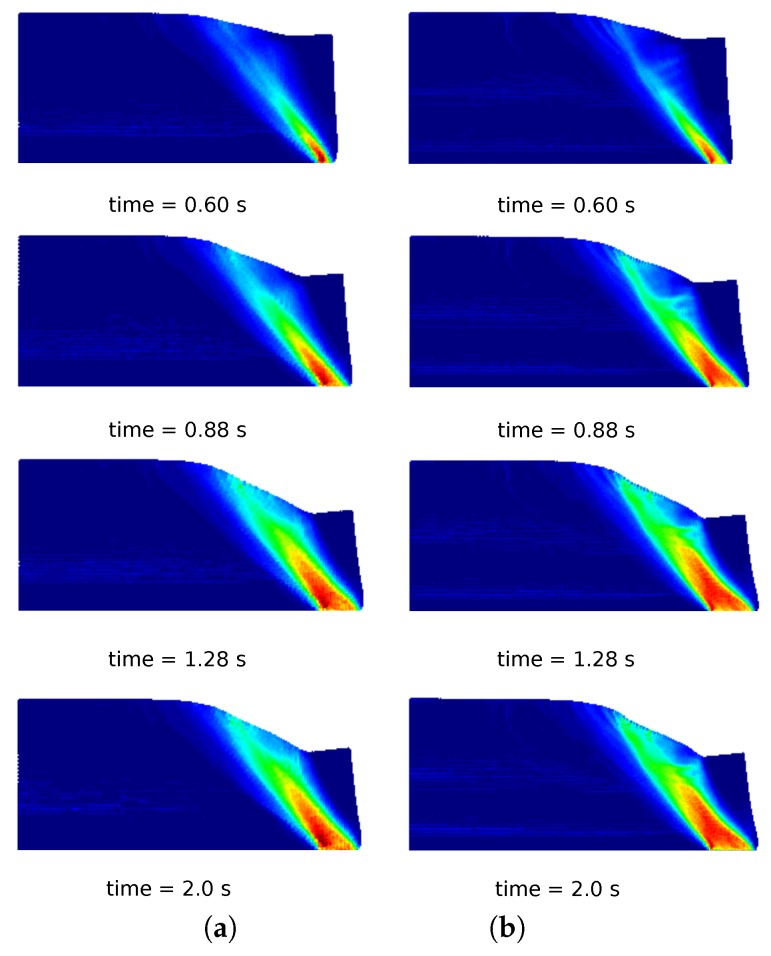
Soil column collapse. Distribution of equivalent plastic strains for different representative time instants in the MPM results. (**a**) Mesh 1; (**b**) Mesh 2.

**Figure 12:**
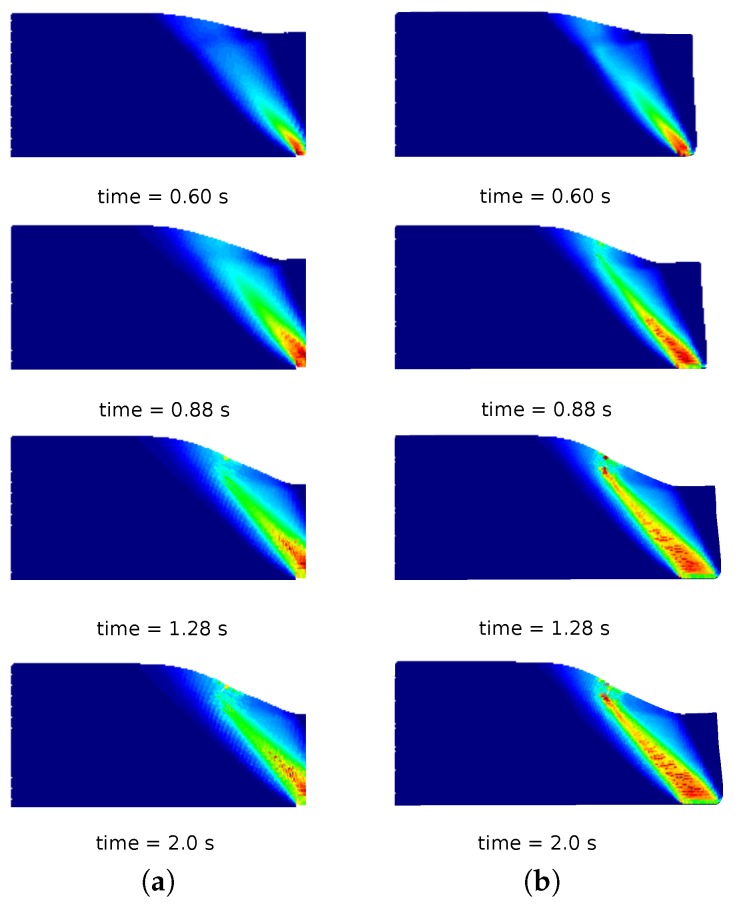
Soil column collapse. Distribution of equivalent plastic strains for different representative time instants in the GMM-MLS results. (**a**) Mesh 1; (**b**) Mesh 2.

**Figure 13:**
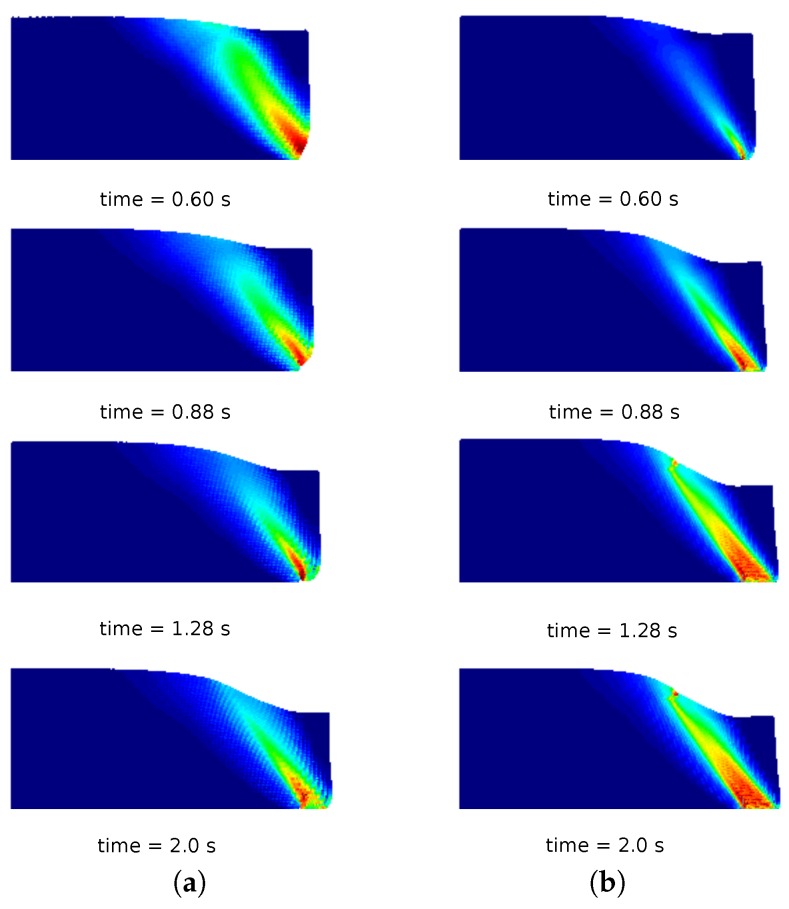
Soil column collapse. Distribution of equivalent plastic strains for different representative time instants in the GMM-LME results. (**a**) Mesh 1; (**b**) Mesh 2.

In Figure 10a, a comparison of the column deformation at different representative time instants is shown. The SPH model taken from [31] predicts a higher final run-out of the column collapse, while the final configurations at time 2.0 s of MPM, GMM-LME and GMM-MLS are almost coincident using Mesh 1 and Mesh 2. It is worth highlighting that GMM-MLS and MPM results of Figure 10 are always in good agreement. However, this is not the case if the evolution of the equivalent plastic strains is observed (see Figure 11 and Figure 12). In the case of GMM-LME, an improvement of the results is noted by using the finer mesh (Mesh 2) in terms of displacements (Figure 10b) and an equivalent plastic strain distribution (Figure 13b). Regarding MPM, it is proven that a good approximation can be obtained using both meshes.

In this example, the capability of handling history-dependent materials, such as cohesive-frictional materials, is verified for both methods. It is noted that, in MPM, large deformations can be naturally tracked without modifying the algorithm, and accurate results are obtained also using the coarser mesh. In GMM, despite the remarkable featrues highlighted in the previous benchmark tests, when the continuum undergoes extremely large deformations, special care should be taken in the definition of the MLS and LME approximants. In this regard, a lack of robustness of the GMM algorithm is observed due to the impossibility of guaranteeing a correct evaluation of the shape functions during the whole deformation process without an ad hoc modification of the procedure for the definition of the connectivity. Thus, for the solution of this example, a correction of the algorithm has been performed and verified to work properly, albeit an increase in the computational time is registered. The establishment of a more general procedure is left for futrue work.

## 6. Conclusions

In the present paper, two particle methods—a material point method and a Galerkin meshless method—are tested and compared to assess their capabilities in solving large displacement and large deformation problems. A variational displacement-based formulation, based on an updated Lagrangian description, is presented, and the algorithms are described in detail.

A comparison of MPM and GMM is performed through three benchmark tests, and the methods are assessed in terms of accuracy, computational time and robustness. The first example is a static cantilever beam. A convergence analysis is performed, and all the techniques have a quadratic convergence rate (compared to an FEM code). Secondly, the dynamic test of a rolling disk on an inclined plane is considered. The robustness of MPM and GMM in dealing with contact between two rigid bodies is tested, and an analysis in terms of computational time and error is performed. We found that GMM, in dynamic cases, has a higher accuracy than MPM, despite a higher computational cost. This is because in MPM, linear basis functions are considered, while in GMM, smooth basis functions are computed allowing one to obtain a superior approximation of the unknown variables. As a last example, a cohesive soil column collapse is analyzed. In this case, we assess the robustness of both methods when the continuum undergoes extremely large deformation. Firstly, it is demonstrated that MPM and GMM can be easily coupled with local plastic laws. Furthermore, it is noted that MPM leads to more accurate results, and the algorithm does not need to be modified in a large deformation case. On the contrary, in GMM, a modification of the algorithm has to be considered to avoid the formation of a non-convex hull of nodes, when the connectivity is defined. Nonetheless, in spite of this modification, a discrepancy in the results is noted, by using either the MLS or the LME technique.

In conclusion, the standard version of MPM represents a good choice to handle problems involving history-dependent materials and large deformations. Regarding GMM, the accuracy of the solution strictly depends on the basis functions chosen. If large deformation of the continuum is not taken into account, this method could be preferred to MPM due to its remarkable featrue in obtaining accurate results in a limited computational time. However, under the finite strain regime, independent of the material to model, the construction of a connectivity in the meshless method becomes more complex, and at least to the authors’ knowledge, a general methodology is still missing to properly define the correct connectivity under any deformation condition. Thus, despite the promising featrues of this approach, an improvement in the robustness of the GMM algorithm is needed to obtain more accurate and reliable solutions in large deformation and failure problems.

## Figures and Tables

**Table 1:** Rolling disk. Absolute errors and computational times.

*Technique*	$\Delta t_{1}$		$\Delta t_{2}$		$\Delta t_{3}$	
		error (m)	$t_{\mathrm{comp}}$ (s)	error (m)	$t_{\mathrm{comp}}$ (s)	error (m)	$t_{\mathrm{comp}}$ (s)
MPM	2.34	232.72	1.27	472.92	0.91	894.91
GMM-MLS	0.84	264.78	0.26	517	0.20	981.82
GMM-LME γ=0.8	1.37	234.32	0.07	460.73	0.06	971.29
GMM-LME γ=1.8	0.9	237.22	0.23	460.50	0.07	1005.21
GMM-LME γ=2.8	0.52	232.42	0.07	466.63	0.06	1038.70
